# Chronic Kidney Disease: Underlying Molecular Mechanisms—A Special Issue Overview

**DOI:** 10.3390/ijms241512363

**Published:** 2023-08-02

**Authors:** Luís Belo, Márcia Carvalho

**Affiliations:** 1 Associate Laboratory i4HB—Institute for Health and Bioeconomy, Department of Biological Sciences, Faculty of Pharmacy, University of Porto, 4050-313 Porto, Portugal; mcarv@ufp.edu.pt; 2 UCIBIO/REQUIMTE, Department of Biological Sciences, Faculty of Pharmacy, University of Porto, 4050-313 Porto, Portugal; 3 FP-I3ID, FP-BHS, University Fernando Pessoa, 4200-150 Porto, Portugal; 4 Faculty of Health Sciences, University Fernando Pessoa, 4200-150 Porto, Portugal

Chronic kidney disease (CKD) is an epidemic health issue that requires global attention [1]. The worldwide increment of CKD incidence is related to an increase in traditional risk factors of the disease (e.g., diabetes, hypertension and human aging) that may be aggravated by other factors, such as environmental and occupational exposure to toxic heavy metals (e.g., cadmium and mercury) [2]. In this Special Issue, authors contributed with works that further the understanding of CKD’s pathophysiology and its associated complications, highlighting the identification of potential new biomarkers of the disease and more effective therapeutic options.

Despite the etiology of CKD, inflammation is a common feature in the pathogenesis of the disease [3]. With this respect, the inflammation pathway driven by tumor necrosis factor (TNF)-α has received particular attention from the scientific community. Several studies in humans and involving animal models report an important role of the TNF system in renal disease [4]. The study of several plasma biomarkers—related to tubular injury, fibrosis and inflammation—revealed that a higher TNF receptor 2 (TNFR2) level was associated with the highest risk of progression of diabetic kidney disease [5]. Also, TNFR2 is distinguished amongst several circulating inflammatory variables because its levels present remarkable negative correlations with estimated glomerular filtration rate (eGFR) and increase with CKD progression [6]. TNF receptors (TNFR1 and TNFR2) might be useful not only for early CKD detection but also as biomarkers of CKD progression and prognosis [4].

New blood and urine biomarkers of CKD have been proposed, but many lack specificity and may only be detected in advanced phases of the disease [7]. Since there are various alternative pathways by which CKD can result, a panel monitoring multiple biomarkers may be a more reasonable method to better anticipate CKD development and access the disease’s outcomes. Modern laboratory methods that enable the simultaneous analysis of several biomarkers are in use and have significantly impacted the identification of new biomarkers. Such is the case of omics technologies, despite being expensive and time-consuming [7]. For instance, Chebotareva and colleagues, by using proteomic analysis, identified potential profile urine biomarkers from patients with focal segmental glomerulosclerosis and minimal change disease, known as podocytopathies clinically manifested by the nephrotic syndrome [8]. Other investigators have used metabolomic studies and microRNA analysis [9].

Altered gut microbiota is involved in the pathogenesis of several pathologies, including kidney diseases [10]. Current knowledge supports the impact of the gut–kidney axis in CKD. A significant qualitative or quantitative alteration in the gut microbiota may lead to reduced production of beneficial bacterial metabolites, such as short-chain fatty acids (SCFAs). SCFAs are known to present important anti-inflammatory properties and their levels are reduced in CKD [11]. Thus, dysbiosis and microbiota metabolite changes may underlie CKD-associated inflammation, promoting disease progression and its associated complications. Recent data also suggest that gut microbiota dysbiosis is implicated in pediatric CKD and renal programming [12]. This programming increases the kidney’s susceptibility to postnatal insults and increases the risk of developing CKD in the future.

The progression of CKD, which ultimately results in an irreversible state of renal fibrosis, is still not well controlled, despite the availability of general treatment methods. Recently, novel drug targets have been proposed: marinobufagenin (a cardiac glycoside) and transient receptor potential cation channel, subfamily C, and member 6 (TRPC6). According to Zheng et al. [13], pharmacological inhibition of TRPC6 may be a viable antifibrotic treatment approach for progressive tubulointerstitial fibrosis in hypertension and metabolic syndrome. Marinobufagenin has been suggested to be involved in profibrotic pathways and to have a relevant role in CKD [14]. Some patients could benefit from therapy with mineralocorticoid receptor antagonists (MRA) that have been shown to have a preventive effect on marinobufagenin-induced fibrosis.

Diuretics are widely used in CKD management, namely in treating edema and hypertension [15]. Diuretics may also affect renal lymphatic vessels, as they express the Na-K-2Cl cotransporter NKCC1, whose function is inhibited by furosemide. Liu and colleagues have demonstrated that lymphatic hyporesponsive to furosemide may occur in situations such as proteinuric kidney disease. Increased renal interstitial sodium after a proteinuric injury is associated with lymphatic vessel dysfunction [16]. 

Musculoskeletal disorders are moderately common complications in CKD patients [17]. CKD-associated cachexia (a syndrome that results in substantial loss of skeletal muscle mass and adipose tissue) has been linked to raised serum levels of pro-inflammatory cytokines, a redox imbalance and growth hormone (GH) resistance [18]. In CKD, some approaches have been proposed to improve muscular wasting, including pharmacological treatment and lifestyle changes. Using an animal model of CKD (mice model consisting of 5/6 nephrectomy), Mak et al. demonstrated that the administration of GH could increase food intake and weight growth, decrease uncoupling proteins and improve muscular mass and function [19]. GH also normalized myogenesis and muscle regeneration. This is in line with studies performed in hemodialysis patients, to whom the administration of GH increased muscle protein synthesis and muscle mass. Compelling evidence also suggests that exercise—a recognized pivotal factor in promoting skeletal muscle remodeling and metabolic adaptation—may help counteract muscle wasting in CKD [20]. Resistance exercise was shown to stimulate protein anabolism in patients under hemodialysis, and endurance exercise improved protein metabolism markers. Intradialytic regular exercise training was also shown to induce a reduction in inflammation and in various redox status parameters while improving physical performance in end-stage renal disease patients [21]. However, global exercise regimens must be implemented to better solidify the outcomes achieved thus far.

In synthesis, the molecular mechanisms involved in the pathophysiology of CKD and associated complications have been explored. The scientific community is also attempting to identify more sensitive and specific biomarkers of CKD but requires further study. Identifying and validating new and standardized therapeutical pharmacological and non-pharmacological options is also mandatory. As guest editors, we thank all the authors and reviewers for their valuable contributions to this issue.
